# Boric Acid Mitigates Alcohol-Induced Renal Podocyte Injury, Apoptosis, and Oxidative Stress in HBV Transgenic Mice

**DOI:** 10.3390/antiox15030318

**Published:** 2026-03-03

**Authors:** Kubra Sevgin, Pelin Erguven, Sevda Tanrikulu-Kucuk, Sevgin Degirmencioglu, Pinar Cetinalp, Soner Aksu, Palmet Gun-Atak, Ibrahim Sogut

**Affiliations:** 1 Department of Histology and Embryology, International Faculty of Medicine, University of Health Sciences, Istanbul 34668, Türkiye; kubra.sevgin@sbu.edu.tr; 2 Department of Biochemistry, Faculty of Medicine, Demiroglu Bilim University, Istanbul 34394, Türkiye; sevda.kucuk@demiroglu.bilim.edu.tr (S.T.-K.); pinarcetinalp@outlook.com (P.C.); 3 Department of Biochemistry, Faculty of Medicine, Kirklareli University, Kirklareli 39060, Türkiye; sevgindegirmencioglu@klu.edu.tr; 4 Department of Molecular Biology and Genetics, Faculty of Engineering and Natural Sciences, Istanbul Health and Technology University, Istanbul 34275, Türkiye; soner.aksu@istun.edu.tr; 5 Medical Biochemistry Laboratory, Liv Hospital, Istanbul 34000, Türkiye; palmetgun@gmail.com

**Keywords:** apoptosis, boric acids, kidney, oxidative stress

## Abstract

Chronic alcohol consumption exacerbates kidney injury, particularly in individuals with hepatitis B virus (HBV) infection. This study investigated the protective effects of boric acid supplementation against alcohol-induced renal damage in HBV transgenic mice. HBV transgenic mice were divided into four groups: control (C), boric acid (B), alcohol (A), and alcohol + boric acid (A + B). Renal injury was evaluated using H&E, PAS, TUNEL, and desmin staining. The expression of caspase-3, cytochrome c, and APAF-1 was analyzed by qRT-PCR. Biochemical analyses included BUN, creatinine, oxidative stress markers (ROS, MDA, TOS, OSI), total antioxidant status, and antioxidant enzyme activities (SOD, CAT, GPx). Histopathological findings showed activated parietal epithelial cells in all groups, indicating renal injury. Alcohol significantly increased tubular damage, podocyte desmin expression, apoptosis, cytochrome c and APAF-1 mRNA levels, and oxidative stress markers, while reducing antioxidant enzyme activities and BUN levels compared with controls. Boric acid supplementation significantly mitigated alcohol-induced tubular injury, apoptosis, oxidative stress, and serum creatinine levels, and improved BUN values. Boric acid treatment alone also alleviated glomerular and tubular injury and reduced tubular apoptosis compared with HBV control mice. Overall, boric acid exerts renoprotective effects in HBV-transgenic mice subjected to chronic alcohol exposure by inhibiting oxidative stress, apoptosis, and podocyte injury.

## 1. Introduction

Hepatitis B virus (HBV) affects nearly 296 million individuals worldwide and remains a leading cause of cirrhosis and hepatocellular carcinoma [1]. Beyond its hepatic consequences, HBV is associated with renal pathologies, including glomerulonephritis, membranous nephropathy, and progressive kidney failure [2,3]. Chronic kidney injury has been reported in up to one-third of patients with advanced cirrhosis [4], and HBV-associated glomerulonephritis (HBV-GN) has been identified in specific patient groups [4,5]. Moreover, untreated HBV infection has been linked to a higher likelihood of developing end-stage renal disease (ESRD) [6]. Management of HBV-related nephritis focuses on reducing viral load; corticosteroids can alleviate proteinuria; however, they carry a risk of HBV reactivation. Antiviral therapies, including interferon-α and nucleos(t)ide analogues, show therapeutic potential, but optimal treatment strategies remain undefined [7].

Chronic alcohol consumption, on the other hand, has been linked to oxidative stress, inflammation, tubular injury, and electrolyte imbalances in the kidney [8]. Similarly, a systematic review and meta-analysis indicated that alcohol misuse is associated with an increased risk of kidney injury, highlighting the need for careful monitoring in affected individuals [9]. Experimental studies have indicated that alcohol may directly exacerbate renal damage through mitochondrial dysfunction, oxidative stress, and inflammation [10]. Although direct investigations on the combined impact of HBV infection and chronic alcohol consumption on kidney function are limited, studies in patients with acute-on-chronic liver disease (AoCLD) indicate that concomitant HBV infection and alcohol exposure worsen liver-related outcomes, implying potential renal consequences [11]. Despite the potential for synergistic mechanisms accelerating kidney damage, the mechanisms underlying the interaction between alcohol and hepatitis B are not fully understood, and the clinical characteristics and outcomes of such complex cases have not been clearly described. In particular, research on the pathophysiology of disease progression in the context of combined HBV infection and alcohol exposure is lacking [11]. Given these individual and combined risks, further research is warranted to explore the synergistic effects of HBV infection and chronic alcohol consumption on kidney function.

Boron is an essential trace element for plants and is believed to play important roles in microorganisms, animals, and humans based on molecular, biochemical, and nutritional evidence [12]. Among its major forms, boric acid (H_3_BO_3_) is a weak Lewis acid (pKa 9.25) that has been widely studied and discussed in recent years for its medical and non-medical applications [13,14]. Boric acid has a plasma half-life of approximately 21 h and is excreted unchanged by the kidneys, remaining detectable in urine for up to 96 h [15]. In addition, it has been demonstrated that at low doses, boric acid enhances antioxidant activity, thereby protecting cell membranes [16,17]. Recent experimental studies have shown that boric acid exerts protective effects against oxidative stress and inflammation while reducing cellular apoptosis. In rat models, it has been reported to mitigate oxidative stress in the kidneys following chronic alcohol exposure [8]. However, to date, no findings have been reported in the literature regarding the effects of boric acid on apoptotic gene expression and histopathological alterations in the renal tissues of HBV transgenic mice subjected to chronic alcohol exposure.

Despite these separate lines of evidence, there is a gap in the literature regarding combined HBV infection and chronic alcohol exposure in kidney tissue, especially in transgenic animal models. Recently, we have presented evidence that boric acid can protect against hepatotoxicity associated with both HBV infection and chronic alcohol consumption [18]. Specifically, there is limited data on how HBV gene expression, renal histopathology, and genetic markers respond when subjected to chronic alcohol, and whether boric acid could offer renal protection in that setting. Therefore, this study aims to investigate the biochemical, histological, and genetic alterations in the kidneys of HBV transgenic mice following chronic alcohol administration, and to explore the potential protective role of boric acid.

## 2. Materials and Methods

### 2.1. Animals

Thirty-two male HBV transgenic mice (CB6F1), eight weeks of age and weighing 20–25 g, were procured from the Experimental Animal Center of TÜBİTAK MRC GEBİ, Türkiye, for use in the in vivo experiments. These mice had been generated by incorporating the HindIII-SacI fragment excised from the pT-HBV1.3 plasmid carrying the complete HBV genome [19]. The presence of HBV DNA in mouse serum samples was confirmed, thereby validating the transgenic model [18]. Animals were provided a standard chow diet and housed in conventional cages under controlled environmental conditions (temperature: 20–22 °C, relative humidity: 50–60%, 12 h light/dark cycle). All experimental procedures were performed in accordance with the ethical standards and approved protocols of the TÜBİTAK Marmara Research Center (MRC) Ethics Committee (approval number: 82080155-7865), approved on 13 October 2025.

### 2.2. Experimental Groups

HBV transgenic mice were randomly divided into four groups (*n* = 8 each): Control (C), Boric Acid (B), Alcohol (A), and Alcohol + Boric Acid (A + B). Considering dextrose solution as a caloric substitute for alcohol (1 g of ethanol = 7.2 kcal, 1 g of dextrose = 3.4 kcal) [20,21], the Control group received an isocaloric dextrose solution (0.5 mL in physiological saline, PSS) by daily gavage for one month. Group B was administered 50 mg/kg/daily boric acid in an isocaloric dextrose solution for 30 days. Group A received ethanol via gavage following a stepwise protocol adapted from established models of chronic alcohol intake [22]: 15% ethanol (1.5 g/kg/day) in week 1, 30% ethanol (3 g/kg/day) in week 2, and 45% ethanol (6 g/kg/day) during weeks 3–4. Group A + B received the same ethanol regimen combined with 50 mg/kg boric acid. After 30 days, mice were anesthetized intraperitoneally with ketamine (100 mg/kg) and xylazine (10 mg/kg) [23], followed by euthanasia via exsanguination. Blood was collected intracardially, and the kidneys were excised. Samples were either snap-frozen in liquid nitrogen for biochemical assays or fixed in neutral formalin (24–48 h) for histopathological evaluation.

### 2.3. Histologic Analysis

Kidney tissues from all animals were fixed in 10% neutral formalin. After standard histologic tissue processing procedures, 5 μm tissue sections were taken from the paraffin tissue blocks. Sections of 5 μm thickness were prepared and stained with hematoxylin and eosin (H&E) as well as periodic acid–Schiff (PAS) for detailed evaluation of tubular, glomerular, and interstitial structures. Renal tissue injury was assessed semi-quantitatively according to the criteria summarized in Table 1. Briefly, tubular injury was scored based on brush border integrity, basement membrane changes, polarity disruption, and the extent of necrosis. Glomerular injury was evaluated by glomerular basement membrane (GBM) alterations, mesangial expansion, and activated parietal epithelial cell (aPEC) hypertrophy/hyperplasia. Tubulointerstitial damage was scored according to the degree and distribution of interstitial inflammation and congestion at 40× magnification. Histological scoring and TUNEL quantification were performed blinded to the treatment group. No additional exclusion criteria were applied beyond technical issues that precluded reliable evaluation. For each animal, 20 randomly selected glomeruli and corresponding cortical microscopic fields were evaluated, and semi-quantitative scores were calculated as the mean value per animal prior to group-level statistical analysis. Each category was graded on a 0–3 scale, where 0 indicated no damage, and 3 represented severe pathological changes (Table 1).

#### 2.3.1. Assessment of Podocyte Injury by Desmin Immunohistochemistry

Immunohistochemical staining for desmin was performed on 4 µm-thick paraffin-embedded kidney sections to assess podocyte injury [24,25]. Following deparaffinization and rehydration, antigen retrieval was carried out in citrate buffer (pH 6.0). Endogenous peroxidase activity was blocked, and sections were incubated overnight at 4 °C with a primary desmin antibody. After incubation with a biotinylated secondary antibody and streptavidin–peroxidase complex, staining was visualized using an AEC chromogen and counterstained with Mayer’s hematoxylin. Negative controls were prepared by omitting the primary antibody. Images were obtained using a light microscope, and for each specimen, 20 randomly selected glomeruli were evaluated at 40× magnification. Desmin staining intensity was scored on a scale from 0 (no staining) to 4 (strong staining) [26], and mean scores were used for statistical analysis.

#### 2.3.2. Detection of Renal Cell Apoptosis by TUNEL Staining

The detection of apoptotic cells in kidney tissue was accomplished through the implementation of the terminal deoxynucleotidyl transferase dUTP nick end labeling (TUNEL) method. For the apoptosis assay, 5 µm sections were stained with terminal transferase-mediated dUTP nick end-labeling reagent (E-CK-A321, Elabscience, Wuhan, China) for in situ apoptosis detection. In brief, 5 µm sections were treated with 20 mg/mL proteinase K and then incubated in a nucleotide mixture containing fluorescein-12-dUTP and terminal deoxynucleotidyl transferase. Positive controls were pretreated with 1 U/mL DNase, and negative controls were incubated without terminal deoxynucleotidyl transferase. Stained sections were examined under a light microscope (Scope.A1; Carl Zeiss, Oberkochen, Germany) equipped with a camera (Axiocam 105 Color; Carl Zeiss, Jena, Germany). Apoptotic cell counts were performed in 20 randomly selected glomeruli and surrounding tubular areas from each tissue sample. Results for apoptotic glomerular cells were expressed as average numbers of TUNEL-positive cells per glomerulus. Results for apoptotic glomerular cells were expressed as average numbers of TUNEL-positive cells per glomerulus [25].

### 2.4. Detection of Renal Cell Apoptosis by RT-PCR

mRNA expression of Caspase-3, Cytochrome-c, and APAF-1 was analyzed in kidney tissues from the experimental groups. Total RNA was extracted from ~50 mg of frozen tissue using RNAzol RT (Molecular Research Center, Cincinnati, OH, USA) following the manufacturer’s protocol. RNA concentration and purity were assessed with a NanoDrop 2000 (Thermo Scientific, Waltham, MA, USA), and samples were normalized prior to cDNA synthesis using the Script cDNA Synthesis Kit (Jena Bioscience, Jena, Germany). Quantitative real-time PCR was performed with qPCR GreenMaster with UNG Kit (Jena Bioscience, Jena, Germany) on a CFX-96 Real-Time PCR System (Bio-Rad, Hercules, CA, USA) using the following cycling conditions: 50 °C for 2 min, 95 °C for 2 min, then 35 cycles of 95 °C for 15 s, 56 °C for 20 s, and 72 °C for 30 s. Relative expression levels were calculated using the ΔΔCT method ((2^−∆∆CT^), with GAPDH as the reference gene. Primers were obtained from LGC Biosearch Technologies (Novato, CA, USA).

### 2.5. Biochemical Measurements of Serum Samples and Tissue Homogenates

Kidney tissues were homogenized in ice-cold 0.15 M KCl (10% *w*/*v*) and centrifuged at 600× *g* for 10 min at 4 °C. The resulting supernatants were used to measure malondialdehyde (MDA), reactive oxygen species (ROS), total antioxidant status (TAS), total oxidant status (TOS), oxidative stress index (OSI), and catalase (CAT) activity. Post-mitochondrial fractions were further centrifuged at 10,000× *g* for 20 min to assess superoxide dismutase (SOD) and glutathione peroxidase (GPx) activities. MDA was determined colorimetrically at 532 nm and expressed as nmol/mg protein [27], while ROS levels were measured fluorometrically (RFU) [28]. TAS, TOS, and OSI (TOS/TAS ratio) were quantified using commercial kits (Rel Assay Diagnostics, Gaziantep, Turkey) [29]. SOD activity was assessed via riboflavin-sensitized photo-oxidation of o-dianisidine at 460 nm and expressed as U/mg protein [30]. CAT activity was determined spectrophotometrically at 240 nm using H_2_O_2_ as a substrate, with one unit defined as the enzyme degrading 1 μmol H_2_O_2_/min at 25 °C. GPx activity was measured at 37 °C using cumene hydroperoxide, with NADPH consumption at 340 nm quantified using its extinction coefficient (6.22 × 10^3^ M^−1^cm^−1^) and expressed as mmol/mg protein [31].

Serum blood urea nitrogen (BUN) and creatinine levels were measured in all experimental groups using enzymatic methods with a commercial Roche kit on a Roche-Hitachi Cobas c311 analyzer (Roche Diagnostics International AG, Rotkreuz, Switzerland).

### 2.6. Statistical Analysis

Statistical analyses of biochemical and histopathological data were performed using SPSS software (version 22.0). The normality of data distribution was assessed using the Kolmogorov–Smirnov and Shapiro–Wilk tests. Data are expressed as mean ± standard deviation (SD) for all variables. For variables that were normally distributed, comparisons among multiple groups were conducted using one-way analysis of variance (ANOVA) followed by Tukey’s post hoc test. For non-normally distributed data, the Kruskal–Wallis test was employed, with Dunn’s multiple comparison test applied for post hoc analysis. Differences were considered statistically significant at *p* < 0.05.

## 3. Results

### 3.1. Histopathological Evaluation

**Figure 1:**
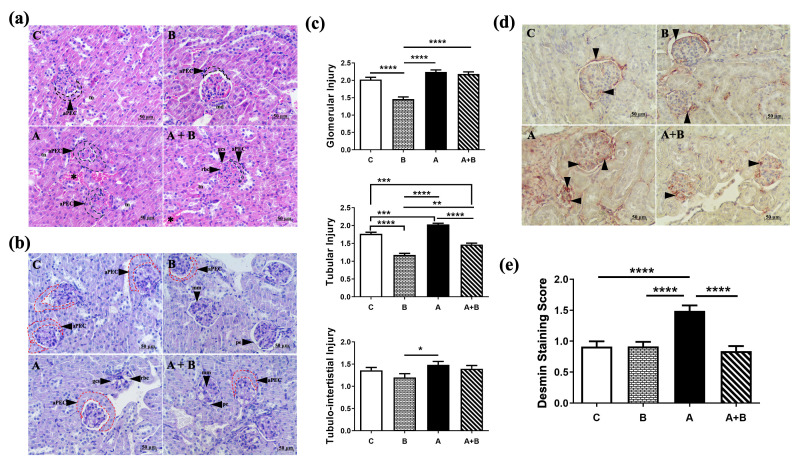
(**a**) HE staining, (**b**) PAS staining of kidney sections. (**c**) Semi-quantitative assessment of glomerular, tubular, and tubulointerstitial injury. Star: congestion in the tubulointerstitial area; aPEC: atypical parietal epithelial cells in red dashed line; pe: parietal epithelium; gbm: glomerular basement membrane; gcs: glomerular capillary system; rbc: red blood cell; tn: tubular necrosis; md: macula densa (*n* = 8) (**d**) Representative desmin IHC staining to demonstrate podocyte injury (black arrowhead: desmin positive cells), 400X magnification (*n* = 8). (**e**) Semi-quantitative assessment of podocyte injury in desmin-stained preparations (*n* = 8). Data were non-normally distributed and analyzed using Kruskal–Wallis test followed by Dunn’s multiple comparisons test. C: control, B: Boric acid, A: Alcohol, A + B: Alcohol + Boric acid group. *p* < 0.05 *, *p* < 0.01 **, *p* < 0.001 ***, *p* < 0.0001 ****.

Although HE and PAS staining revealed varying degrees of kidney damage among the experimental groups (Figure 1a,b), a common histopathological feature observed in all groups was the presence of activated parietal epithelial cells (dashed lines; aPECs), characterized by proliferation and hypertrophy of the glomerular parietal epithelium. The mesangial matrix density appeared similar across the groups; however, pronounced dilation of the glomerular capillary system (gcs) was particularly evident in the alcohol (A) group. Semi-quantitatively, the alcohol (A) group exhibited marked tubular (*p* < 0.0001), glomerular (*p* < 0.0001), and tubulointerstitial damage (*p* < 0.05) compared to the boric acid (B) group. Boric acid alone (B) effectively reduced both glomerular and tubular injury (*p* < 0.0001) relative to the control (C) group, indicating a potential protective baseline effect. In the combined treatment group (A + B), glomerular (*p* < 0.0001) and tubular (*p* < 0.01) injury scores were higher than those observed in the boric acid group (B), with more pronounced glomerular and tubular alterations. However, among alcohol-treated animals, tubular injury was significantly reduced in the A + B group compared with both the alcohol (A) (*p* < 0.0001) and control (C) groups (*p* < 0.001), suggesting a partial protective effect of boric acid against alcohol-induced tubular damage. Semi-quantitative scoring supported these observations (Figure 1c). Detailed intergroup comparisons are provided in Table S1.

Desmin immunostaining demonstrated significantly increased desmin-positive podocytes in the alcohol (A) group compared to other groups (*p* < 0.0001), consistent with podocyte injury (Figure 1d). Boric acid (B) treatment to alcohol administered mice in the A + B group demonstrated a decrease in podocyte injury by desmin immunolabeling (Figure 1e, *p* < 0.0001). Semi-quantitative analysis confirmed these patterns (Figure 1e).

### 3.2. Apoptosis in Glomerular and Tubular Cells

**Figure 2:**
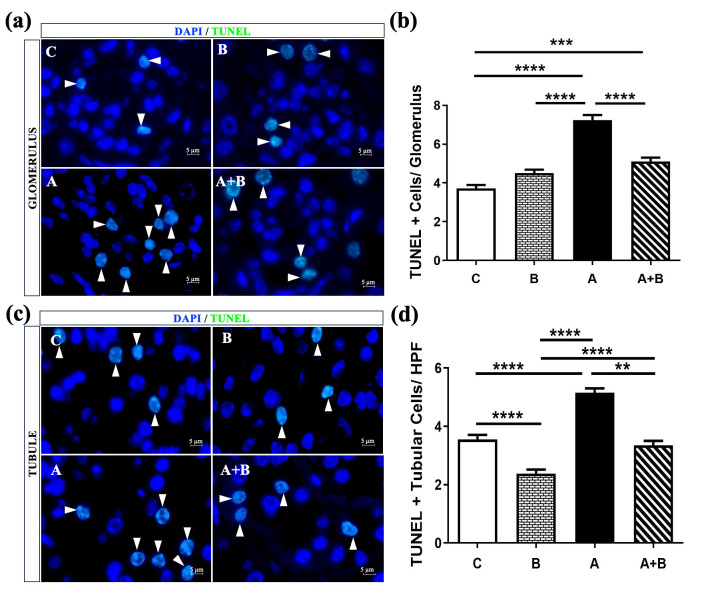
(**a**) Representative TUNEL staining of glomerular (**a**) and tubular (**c**) cells (White arrowhead: TUNEL+ cell). (**b**) Semiquantitative analysis of TUNEL+ cells per glomerulus (*n* = 8). (**b**) and tubular area (**d**) per high power field (HPF) at 100X magnification. Data were non-normally distributed and analyzed using Kruskal–Wallis test followed by Dunn’s multiple comparisons test. C: Control, B: Boric acid, A: Alcohol, A + B: Alcohol +Boric acid group. *p* < 0.01 **, *p* < 0.001 ***, *p* < 0.0001 ****.

Staining demonstrated increased apoptotic cell counts in both glomerular and tubular compartments of the alcohol (A) group compared with all other groups (Figure 2a,d). Semi-quantitative analysis confirmed a significant elevation in the number of TUNEL-positive cells per glomerulus and per high-power field (HPF) in the tubular area (*p* < 0.001–0.0001) (Figure 2b). In the A + B group, glomerular apoptosis remained significantly higher than in the control (C) group (*p* < 0.001), whereas tubular apoptosis was markedly reduced compared with the alcohol (A) group (*p* < 0.01), indicating a partial protective effect of boric acid. Boric acid alone (B) significantly decreased tubular apoptosis relative to the control (*p* < 0.0001), suggesting an intrinsic anti-apoptotic influence. However, among boric acid–treated groups, the A + B group showed a significantly higher number of apoptotic tubular cells than the B group (*p* < 0.0001), reflecting incomplete protection under alcohol exposure.

### 3.3. Expression of Apoptosis-Related Genes

**Figure 3:**
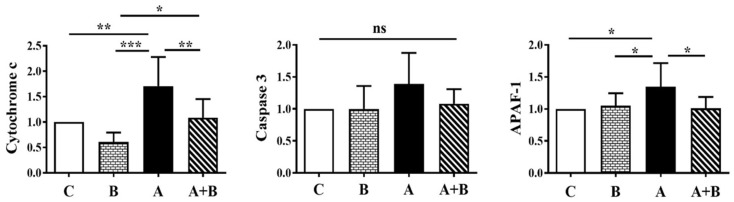
Cytochrome c, caspase 3, and APAF-1 mRNA expression in experimental groups. Caspase-3 data were non-normally distributed and analyzed using the Kruskal–Wallis test with Dunn’s post hoc test, whereas Cytochrome c and APAF-1 data were normally distributed and analyzed by one-way ANOVA with Tukey’s post hoc test. C: Control, B: Boric acid, A: Alcohol, A + B: Alcohol +Boric acid group (*n* = 8). *p* < 0.05 *, *p* < 0.01 **, *p* < 0.001 ***, ns: non-significant.

Renal mRNA expression levels of cytochrome c, caspase-3, and APAF-1 were evaluated across experimental groups (Figure 3). Caspase-3 expression did not differ significantly among the groups (ns). In contrast, cytochrome c and APAF-1 mRNA levels were significantly elevated in the alcohol (A) group compared with all other groups (*p* < 0.05–0.001). Boric acid co-treatment (A + B) reduced both cytochrome c (*p* < 0.01) and APAF-1 (*p* < 0.05) expression relative to the alcohol (A) group, indicating attenuation of alcohol-induced mitochondrial apoptotic signaling. When comparing the boric acid–treated groups, cytochrome c expression remained significantly higher in the A + B group than in the B group (*p* < 0.05), suggesting incomplete protection under alcohol exposure.

### 3.4. Evaluation of Oxidative Stress and Renal Biochemical Parameters

**Figure 4:**
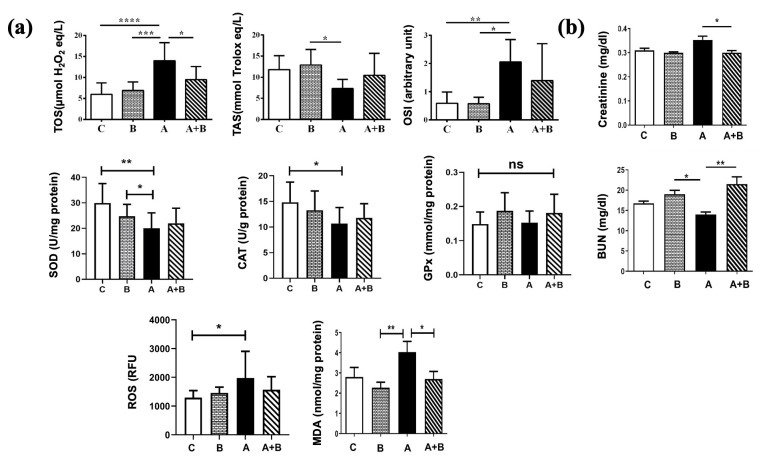
(**a**) TAS, TOS, OSI, MDA, ROS levels, and GPx, SOD, CAT activities of kidney tissue samples between experimental groups (*n* = 8). (**b**). Creatinine and BUN levels of serum samples in experimental groups (*n* = 8). All data, except GPx, were analyzed using the Kruskal–Wallis test followed by Dunn’s multiple comparison post hoc test. GPx was analyzed using one-way ANOVA following Tukey’s multiple comparisons test. C: Control, B: Boric acid, A: Alcohol, A + B: Alcohol +Boric acid group. *p <* 0.05 *, *p <* 0.01 **, *p* < 0.001 ***, *p* < 0.0001 ****, ns: non-significant.

TOS level was significantly higher in the alcohol (A) group compared to all groups (*p* < 0.05). TAS level was significantly lower in the alcohol (A) group, and OSI level was significantly higher in the alcohol (A) group compared to the boric acid (B) group (*p <* 0.05). This increase in OSI was also higher compared to the control group (*p <* 0.01). Decrease in SOD (*p <* 0.01) and CAT (*p <* 0.05) activity was also significant compared to control. Decreased SOD activity was also significant compared to the boric acid (B) group (*p <* 0.05). GPx activity did not differ significantly among the groups. ROS level was higher in the alcohol (A) group compared to the control (*p <* 0.05). Tissue MDA level significantly increased in the alcohol (A) group compared to the boric acid group (*p <* 0.01), which was also significanly decrased significantly decreased with boric acid administration in the A + B group (*p <* 0.05). Among the alcohol administered groups, boric acid significantly decreased the serum creatinine level (*p <* 0.05) and increased the BUN levels (*p <* 0.01) compared to the alcohol (A) group. A decrease in serum BUN level was also significant compared to the boric acid (B) group (*p <* 0.05) (Figure 4).

## 4. Discussion

In HBV transgenic mice, chronic alcohol exposure induced pronounced tubular injury, increased desmin expression, activation of mitochondrial apoptotic mediators (cytochrome c, APAF-1), enhanced glomerular and tubular apoptosis, elevated oxidative stress markers, and reduced antioxidant enzyme activities. Boric acid treatment markedly attenuated these changes by restoring antioxidant balance, reducing lipid peroxidation, and suppressing apoptosis. Moreover, boric acid alone reduced glomerular and tubular injury and tubular cell apoptosis, indicating its intrinsic renoprotective potential.

Immune complex deposition, particularly involving low-molecular-weight HBeAg capable of crossing the glomerular basement membrane to form subepithelial deposits, is a key mechanism underlying HBV-associated nephropathy, leading to both glomerular and tubular injury [32]. Studies showed that HBV-associated glomerulopathy may be associated with the deposition of HBV-associated antigens such as HBeAg and IgG antibodies [3]. In addition, it has been shown that activation of parietal epithelial cells (PECs) represents a proliferative response that contributes to glomerular remodeling and fibrosis [33]. In the present study, HBV and/or chronic alcohol exposure may have similarly induced PEC activation or promoted epithelial–mesenchymal transition (EMT), a process contributing to fibrogenic and maladaptive repair mechanisms [33,34]. Chronic alcohol consumption in HBV transgenic mice (A group) resulted in marked renal damage characterized by glomerular, tubular, and tubulointerstitial injury, accompanied by elevated desmin expression—an indicator of podocyte stress and glomerular barrier disruption [35]. The presence of desmin-positive PECs across all experimental groups suggests the involvement of activated PECs (aPECs) in glomerular repair and their potential to differentiate into podocytes [33,36]. Administration of boric acid alone (B group) caused minimal histopathological alterations and mitigated glomerular and tubular injury compared with HBV+ controls, supporting its intrinsic renoprotective properties [8,37]. Co-administration of boric acid with alcohol (A + B group) significantly alleviated tubular and podocyte injury, as evidenced by reduced desmin labeling, although glomerular injury persisted, indicating partial protection. This partial effect may be attributed to the selective antioxidant actions of boric acid in renal tubules [38], MMP-7–mediated nephrin degradation [39], and ethanol-induced loss of podocyte structural proteins such as nephrin and podocin [39]. Collectively, these findings demonstrate that boric acid markedly enhances tubular resilience and preserves podocyte integrity even under chronic alcohol exposure. Its potential role in mitigating MMP-7–mediated podocyte injury [40] and modulating PEC-driven glomerular remodeling under HBV and alcohol exposure warrants further investigation.

Beyond structural and phenotypic alterations, renal injury in HBV transgenic mice may also involve apoptosis as a key mechanism of cell loss and tissue remodeling [41]. In this context, we evaluated the apoptosis to provide further insight into the cytoprotective effects of boric acid against HBV- and alcohol-induced renal damage. Studies on mice show that HBV infection can elevate the inflammation, decrease antioxidant enzyme expression, and increase mitochondrial dysfunction [42]. The combination of these factors has been shown to hasten the progression of liver disease in HBV-infected individuals [43]. Deng et al. showed that excessive apoptosis of renal proximal tubular cells may be associated with renal injury in patients with chronic HBV infection [44]. Alcohol further triggers apoptosis via intrinsic, extrinsic, and caspase-independent pathways, with mitochondrial cytochrome c release activating APAF-1 and caspase-9, ultimately leading to caspase-3–mediated cell death [20,45,46,47]. In this study, boric acid, which can modulate apoptotic gene expression through Bcl-2, Bax, and caspase-3, calcium signaling, MAPK pathways, and p53 regulation [48,49], was hypothesized to reduce apoptosis under HBV and alcohol-induced oxidative stress [17,20]. Consistently, in the present study, boric acid decreased cytochrome c and APAF-1 expression and TUNEL-positive cells in alcohol-exposed HBV mice, demonstrating anti-apoptotic effects. Boric acid alone also reduced tubular apoptosis, confirming its intrinsic anti-apoptotic and cytoprotective properties in HBV-infected tissue. However, persistent TUNEL positivity and elevated cytochrome c in the A + B group indicate that alcohol partially counteracts this protection, while unchanged caspase-3 suggests modulation may also occur upstream in mitochondrial signaling or via caspase-independent pathways. Overall, boric acid exerted partial but notable protection against HBV + alcohol-induced renal apoptosis by attenuating ROS-driven mitochondrial dysfunction and stabilizing early apoptotic signaling.

The biochemical findings complemented these histopathological and molecular observations. Alcohol exposure induced a strong oxidative stress response in HBV transgenic mice, characterized by elevated TOS and OSI levels and reduced TAS, SOD, and CAT activities, which may be partly due to impaired podocyte function, as these cells express alcohol dehydrogenase (ADH) and cytochrome P450 (CYP450) hydroxylase that normally maintain glomerular barrier integrity through 20-Hydroxyeicosatetraenoic acid (20-HETE) production, but are disrupted by high ethanol or ADH inhibition, increasing protein permeability [39]. Maintenance of redox homeostasis is fundamental to cellular integrity, as tightly regulated ROS levels support physiological signaling, whereas excessive ROS accumulation triggers oxidative stress, lipid peroxidation, mitochondrial dysfunction, and apoptotic cell death [50,51]. Disturbances in redox balance are closely linked to renal injury through tubular and podocyte damage and activation of mitochondrial apoptotic signaling [52]. Enzymatic antioxidant systems, including SOD, CAT, and GPx, are central to protecting renal tissue from oxidative insults [53], highlighting redox modulation as a potential therapeutic target in alcohol- and HBV-associated nephropathy. Accordingly, boric acid markedly improved redox status by decreasing MDA and ROS, restoring SOD and CAT activity, and reducing OSI, particularly in the A + B group, thereby limiting lipid peroxidation and reinforcing antioxidant defenses, despite unaltered GPx activity. Although not included in the study scope, alcohol-induced activation of HBV transcription [54] and its oxidative effects on the kidney [8,9] may exacerbate tissue damage in the alcohol group, which appears alleviated by the antioxidant properties of boric acid [8,12,38,55]. In line with the present study, previous experimental studies have shown that boric acid exerts antioxidant and anti-apoptotic effects against alcohol-induced renal injury. In mice exposed to chronic ethanol, boric acid treatment significantly reduced Caspase-3 expression and TUNEL-positive cells, decreased MDA, TOS, and OSI levels, and increased TAS, indicating suppression of oxidative stress–mediated apoptosis [38]. Similarly, in acute ethanol toxicity, boric acid (50–100 mg/kg) inhibited Caspase-3 activity, enhanced SOD, CAT, and GPx enzymes, and mitigated renal oxidative damage [8]. Another study confirmed reduced TUNEL positivity and tubular injury in boric acid-treated rats [56]. Functionally, boric acid co-treatment partially restored renal function, lowering serum creatinine and increasing BUN relative to alcohol alone, consistent with the observed histological, molecular, and biochemical protection. Reduced BUN in the alcohol group (A) likely reflects impaired hepatic urea synthesis [57], while its increase with boric acid may result from improved glomerular filtration or fluid balance [58].

This study provides novel experimental evidence on the potential protective role of boric acid in HBV transgenic mice with chronic alcohol exposure, highlighting its antioxidative and renoprotective effects. The integration of biochemical, histopathological, and immunohistochemical analyses strengthens the reliability of the findings. Importantly, the use of HBV transgenic mice provides a clinically relevant model, as all animals carry HBV and thus exhibit increased renal susceptibility, mimicking the human condition. Although boric acid did not markedly affect some renal parameters in HBV transgenic mice without alcohol exposure, its protective effects became evident under chronic alcohol stress, demonstrating its potential to mitigate alcohol-induced renal injury in a susceptible kidney context. However, several limitations should be acknowledged. The HBV transgenic mouse model may not fully recapitulate human HBV pathophysiology. In addition, the absence of a healthy (non-HBV) control group limited direct comparison with baseline renal architecture and function. A single boric acid dose and treatment duration were employed, limiting assessment of dose-dependent effects. The mechanisms underlying incomplete glomerular recovery—such as immune complex deposition, local inflammation, and HBV antigen persistence—were not explored in depth. Finally, the potential impact of boric acid on HBV replication and HBV-driven inflammation remains uncertain; future studies assessing serum HBV DNA, HBsAg/HBeAg levels, and hepatic viral markers would be necessary to distinguish HBV-independent renal effects.

## 5. Conclusions

In conclusion, this study provides experimental evidence demonstrating that boric acid exerts both structural and biochemical protection against alcohol-induced renal injury in an HBV transgenic model, thereby addressing an underexplored interaction between chronic alcohol exposure and HBV-related kidney damage. Its effects were mainly mediated through attenuation of oxidative stress, suppression of mitochondrial apoptotic signaling, and preservation of tubular integrity. However, glomerular protection remained incomplete, likely due to persistent HBV- and alcohol-induced oxidative and apoptotic stress. By highlighting the differential tubular versus glomerular response, the present findings contribute to an understanding of the multifactorial mechanisms underlying HBV-associated renal injury. These findings highlight the multifactorial nature of HBV-related renal injury and suggest that antioxidative strategies, though limited, may offer therapeutic benefit. Importantly, this study establishes a translational framework for evaluating boric acid as a supportive therapeutic approach in HBV-associated nephropathy complicated by alcohol exposure. Therefore, future studies incorporating HBV-negative controls and viral replication markers are warranted to clarify whether boric acid exerts direct antiviral or HBV-specific protective effects beyond its antioxidative properties.

## Figures and Tables

**Table 1:** Tubular, glomerular, and interstitial evaluation of kidney tissue (0: Normal, 1: Mild, 2: Moderate, 3: Severe).

Tissue	Damage	Score
Tubular	Normal: Tubular epithelium intact. Normal brush border and polarity. No hypertrophy, hyperplasia, vacuolization, or basement membrane changes.	0
	Mild: Mild thickening or irregularity of the tubular basement membrane. Focal tubular necrosis affecting < 25. Brush border slightly reduced or patchy	1
	Moderate: Partial loss of brush border and polarity disruption. Focal tubular necrosis affects 26–50% of the tubular area. Basement membrane thickening combined with the above changes	2
	Severe: Brush border is mostly lost. Tubular necrosis affecting > 50% of the cortical area. The tubular lumen often contains protein casts or cellular debris.	3
Glomerular	No damage: Normal glomerular architecture; intact GBM and Bowman’s capsule; no activated parietal epithelial cell (aPEC), hypertrophy/hyperplasia.	0
	Mild: Thickening of Bowman’s capsule and/or GBM. Mild mesangial expansion. Focal aPEC hypertrophy/hyperplasia involving ≤ 25% of Bowman’s capsule.	1
	Moderate: Mesangial matrix proliferation. aPEC hypertrophy/hyperplasia involving 26–50% of Bowman’s capsule, partially narrowing Bowman’s space.	2
	Severe: aPEC hypertrophy/hyperplasia involving > 50% of Bowman’s capsule, causing marked obliteration of Bowman’s space.	3
Tubulointerstitial	No damage: Normal interstitium; no inflammation, hemorrhage,	0
	Mild: Interstitial inflammation and/or congestion affecting < 25% of the glomerular surrounding area.	1
	Moderate: Interstitial inflammation and/or congestion affecting 26–50% of the glomerular surrounding area.	2
	Severe: Interstitial inflammation and/or congestion affecting > 50% of the glomerular surrounding area.	3

## Data Availability

The original contributions presented in this study are included in the article/Supplementary Materials. Further inquiries can be directed to the corresponding author(s).

## Supplementary Materials

The following supporting information can be downloaded at: https://www.mdpi.com/article/10.3390/antiox15030318/s1, Table S1: Statistical comparison among experimental groups with 95% confidence intervals.

## Abbreviations

The following abbreviations are used in this manuscript:

APAF1	Apoptotic Protease-Activating Factor 1
BUN	Blood Urea Nitrogen
CAT	Catalase
GPx	Glutathione Peroxidase
HBV	Hepatitis B Virus
OSI	Oxidative Stress Index
qRT-PCR	Quantitative Real-Time Polymerase Chain Reaction
ROS	Reactive Oxygen Species
SOD	Superoxide Dismutase
TAS	Total Antioxidant Status
TOS	Total Oxidant Status
TUNEL	Terminal Deoxynucleotidyl Transferase dUTP Nick-End Labeling
