# Supplementary material for: Boric Acid Mitigates Alcohol-Induced Renal Podocyte Injury, Apoptosis, and Oxidative Stress in HBV Transgenic Mice
Source: Antioxidants (Basel). 2026 Mar 3;15(3):318. doi: 10.3390/antiox15030318 (PMC13024679; doi:10.3390/antiox15030318)
Supplement: Supplementary file 1 [file antioxidants-15-00318-s001.zip › antioxidants-4140077-supplementary.pdf]

Table S1. Statistical comparison among experimental groups with 95% confidence intervals.

	C	B	A	A+B	Mean Rank Diff.	P	
**Glomerular Injury**	2.0 (1.5-2.5)	1.5 (1.0-2.0)	2.5 (2.0-2.5)	2.4 (2.0-2.5)	91<br>-125.5<br>-115.5	B-C ****<br>B-A****<br>B-A+B ****	Kruskal-Wallis test<br>Dunn's multiple comparisons test
**Tubular Injury**	1.5 (1.5-2.0)	1.0 (1.0-1.5)	2.0 (2.0-2.0)	1.5 (1.5-1.5)	124.9<br>-61.85<br>66.02<br>-186.7<br>-58.87<br>127.9	C-B ****<br>C-A ***<br>C-A+B ***<br>B-A ****<br>B-A+B **<br>A-A+B ****	Kruskal-Wallis test<br>Dunn's multiple comparisons test
**Tubulo-intertistial Injury**	1.0 (1.0-1.5)	1.0 (0.5-2.0)	1.5 (1.0-2.0)	1.5 (1.0-2.0)	-46.12	B-A *	Kruskal-Wallis test<br>Dunn's multiple comparisons test
**TUNEL + Cells/ Glomerulus**	4.0 (3.0-5.0)	4.0 (4.0-5.0)	8.0 (5.0-9.0)	5.0 (4.0-6.0)	-117.5<br>-52.5<br>-89.5<br>65	C-A ****<br>C-A+B ***<br>B-A ****<br>A-A+B ****	Kruskal-Wallis test<br>Dunn's multiple comparisons test
**TUNEL + Tubular Cells/ HPF**	3.5 (3.0-5.0)	2.0 (1.25-3.0)	5.0 (4.0-6.0)	3.0 (3.0-4.0)	49<br>-73.5<br>-122.5<br>-42.5<br>80	C-B ****<br>C-A ****<br>B-A ****<br>B-A+B **<br>A-A+B ****	Kruskal-Wallis test<br>Dunn's multiple comparisons test
**Podocyte Injury by Desmin**	0.5 (0.0-1.5)	1.0 (0.5-2.0)	1.5 (1.0-2.0)	0.5 (0.0-1.0)	-78.4<br>-73.06<br>86.57	C-A ****<br>B-A****<br>A-A+B ****	Kruskal-Wallis test

							Dunn's multiple comparisons test
**MDA**	1.95 (1.825-2.95)	1.8 (1.4-3.4)	3.25(2.3-3.9)	1.8(1.65-4.25)	18.12<br>-21.76	A-A+B *<br>B-A**	Kruskal-Wallis test<br>Dunn's multiple comparisons test
**ROS**	1185(1107-1471)	1469(1442-1604)	1780(1340-1922)	1279(1243-1839)	-22.18	C-A*	Kruskal-Wallis test<br>Dunn's multiple comparisons test
**SOD**	26.7(24.98-37.88)	25.7(19.2-28.7)	18.8(15.5-23.0)	22.6(17.53-26.45)	26.38<br>19.21	C-A ***<br>B-A*	Kruskal-Wallis test, Dunn's multiple comparisons test
**CAT**	14.4(11.05-18.88)	14.8(9.1-16.1)	10.65(10.1-13.4)	11.0(10.58-13.48)	19.65	C-A*	Kruskal-Wallis test, Dunn's multiple comparisons test
**GPx**	0.1488±0.035	0.1871±0.053	0.1530±0.033	0.1811±0.054		ns	One-way ANOVA<br>Tukey’s multiple comparisons test
**BUN**	16.5(15.25-18.5)	18(17-22)	13(13-16)	20.5(17-27)	14.67<br>-16.0	B-A*<br>A-A+B**	Kruskal-Wallis test<br>Dunn's multiple comparisons test

**Creatinine**	0.31(0.28-0.33)	0.3(0.29-0.31)	0.34(0.32-0.4)	0.29(0.28-0.32)	12.92	A-A+B*	Kruskal-Wallis test Dunn's multiple comparisons test
**Cytochrome c**	1.0	0.60±0.19	1.70±0.57	1.08±0.36	-0.704<br>-1.100<br>-0.504<br>0.618	C&A **<br>B&A***<br>B&A+B *<br>A&A+B **	One-way ANOVA Tukey's multiple comparisons test
**Caspase 3**	1.0	1.13 (0.24-1.31)	1.41 (0.60-2.24)	1.14 (0.63-1.29)	-	ns	Kruskal-Wallis test Dunn's multiple comparisons test
**APAF-1**	1.0	1.05±0.19	1.35±0.37	1.01±0.17	-0.348<br>-0.296<br>0.355	C&A *<br>B&A *<br>A&A+B *	One-way ANOVA Tukey's multiple comparisons test

One-Way ANOVA test, Tukey's multiple comparisons test are presented as Mean±SD.Kruskal-Wallis test and Dunn's multiple comparisons test are presented as Median (25% Percentile-75% Percentile). p< 0.05 *, p< 0.01 **, p<0.001 ***, p<0.0001 ****, ns: non significant.
